# Underground electrotonic signal transmission between plants

**DOI:** 10.1080/19420889.2020.1757207

**Published:** 2020-04-28

**Authors:** Alexander G. Volkov, Yuri B. Shtessel

**Affiliations:** a Department of Chemistry, Oakwood University, Huntsville, AL, USA; b Department of Electrical and Computer Engineering, University of Alabama in Huntsville, Huntsville, AL, USA

**Keywords:** *Aloe vera*, *Brassica oleracea* L., electrostimulation, electrotonic potential, plant-soil-plant signaling

## Abstract

Plants can communicate with other plants using wireless pathways above and underground. Some examples of these underground communication pathways are: (1) mycorrhizal networks in the soil; (2) the plants’ rhizosphere; (3) acoustic communication; (4) naturally grafting of roots of the same species; (5) signaling chemicals exchange between roots of plants; and (6) electrical signal transmission between plants through the soil. To avoid the possibility of communication between plants using mechanisms (1)–(5), soils in both pots with plants can be connected by Ag/AgCl or platinum wires. Electrostimulation *Aloe vera* or cabbage plants induces electrotonic potentials transmission in the electro-stimulated plants as well as in the neighboring plants located in the same or different electrically connected pots regardless if plants are the same or different types. The amplitude and sign of electrotonic potentials in both electrostimulated and neighboring plants depend on the amplitude, rise, and fall of the applied voltage. Electrostimulation serves as an important tool for the evaluation of mechanisms of underground communication in the plant-wide web. The previously developed mathematical model of electrotonic potentials transmission within and between tomato plants, which is supported by the experimental data, is generic enough to be used for simulation study and predicting the intercellular and intracellular communication in the form of electrical signals in the electrical networks within and between a variety of plants.

There are many possible pathways for plants underground communication [1–8]: (1) electrical signal transmission [1–4]; (2) mycorrhizal networks in the soil [5]; (3) the plants’ rhizosphere (root ball) [6]; (4) naturally grafting of roots of the same species [7]; (5) chemical signaling between roots of plants [8,9]; (6) acoustic communication [10,11]. Recently we found that there is a ultrafast electrical signal transmission between neighboring plants – fast underground electrical signal propagation between roots through the soil [2,3]. The possibility of electrical communication between plants in different pots connected by a metal conductor to avoid the signal transduction between plants in the same pot through mycorrhizal networks in the soil will be interesting to pursue.

Electrostimulation of plants can induce activation of ion channels and ion transport, gene expression, enzymatic systems activation, electrical signaling, plant movements, enhance wound healing, repair plant-cell damage, and influence plant growth [12-14].

There are five major types of electrical signaling in plants and animals: action potentials, electrotonic potentials, graded potentials, receptor potentials, and streaming potentials. Electrical signals can propagate inside plants along the plasma membrane on short distances in plasmodesmata, and on long distances in a phloem. The action potential can propagate over the entire length of the cell membrane and along the conductive bundles of tissue with constant amplitude, duration, and speed. Electrotonic potentials in plants exponentially decrease with distance [2,3]. A graded potential is a wave of electrical excitation that corresponds to the size of the stimulus. Receptor potentials are generated by mechanosensitive ion channels. A streaming potential is a potential difference that arises across a capillary tube or membrane when an electrolyte solution is forced through it. Streaming potentials exist in plants and in soil.

In small neurons, exponentially decreasing electrical potentials are referred to as electrotonic potentials [15]. Electrotonic potentials exist not only in small neurons but also in plants [2–4]. Electrostimulation of electrical circuits in the Venus flytrap, *Aloe vera, Arabidopsis thaliana, Mimosa pudica*, apple fruits, and potato tubers induce electrotonic potentials with amplitude exponentially decreasing along vascular bundles [2,16]. In the electrical stimulation of the Venus flytrap, the lower leaf induces electrotonic signals within the entire plant. Electrotonic potentials can induce action potentials in plants [16], small neurons, and dendrites [15].

Some authors use in plant electrophysiology additional terminology such as systemic [17,18] and variation potentials [19–21]. Systemic potential can be caused by stimulating the plasma membrane H^+^ -ATPase, which may hold and transport information systemically within the whole plant or at least in parts of the plant [17]. A variation potential was introduced last century [19] for a hydraulically propagating electrical signal in plants like streaming potential in electrocapillary phenomena [12,22].

Plants can detect their neighbor at the root level [23]. Soil is a good electrical conductor for transmission of electrical signals between plants [24]. Physiological role of electrical signals propagating between plants can be very important step in development of knowledge in field of the plant communication [25].

The goal of this work is to find if fast electrical signal conduction exists between different neighboring plants in separate pots connected by electrical conductors without volatile organic compounds’ emission, mycorrhizal networks in the soil, roots grafting, or acoustic communication.

Electrostimulation of a plant by a square pulse from a function generator or a battery induces percussive electrotonic signals along the same plant and in other plants in the same pot (Figure 1). Soil can work as an electrical conductor between roots of plants [1–4,24]. To avoid root-to-root connections or mycorrhizal networks between plants, we connected soil near plants in different pots by a platinum or silver wire. Since soil and plants have electrolyte solutions with Cl^−^ anions, we also used double sided Ag/AgCl electrode. The pulse train, sinusoidal and a triangular saw-shape voltage profiles were used for electrostimulation.

**Figure 1.**
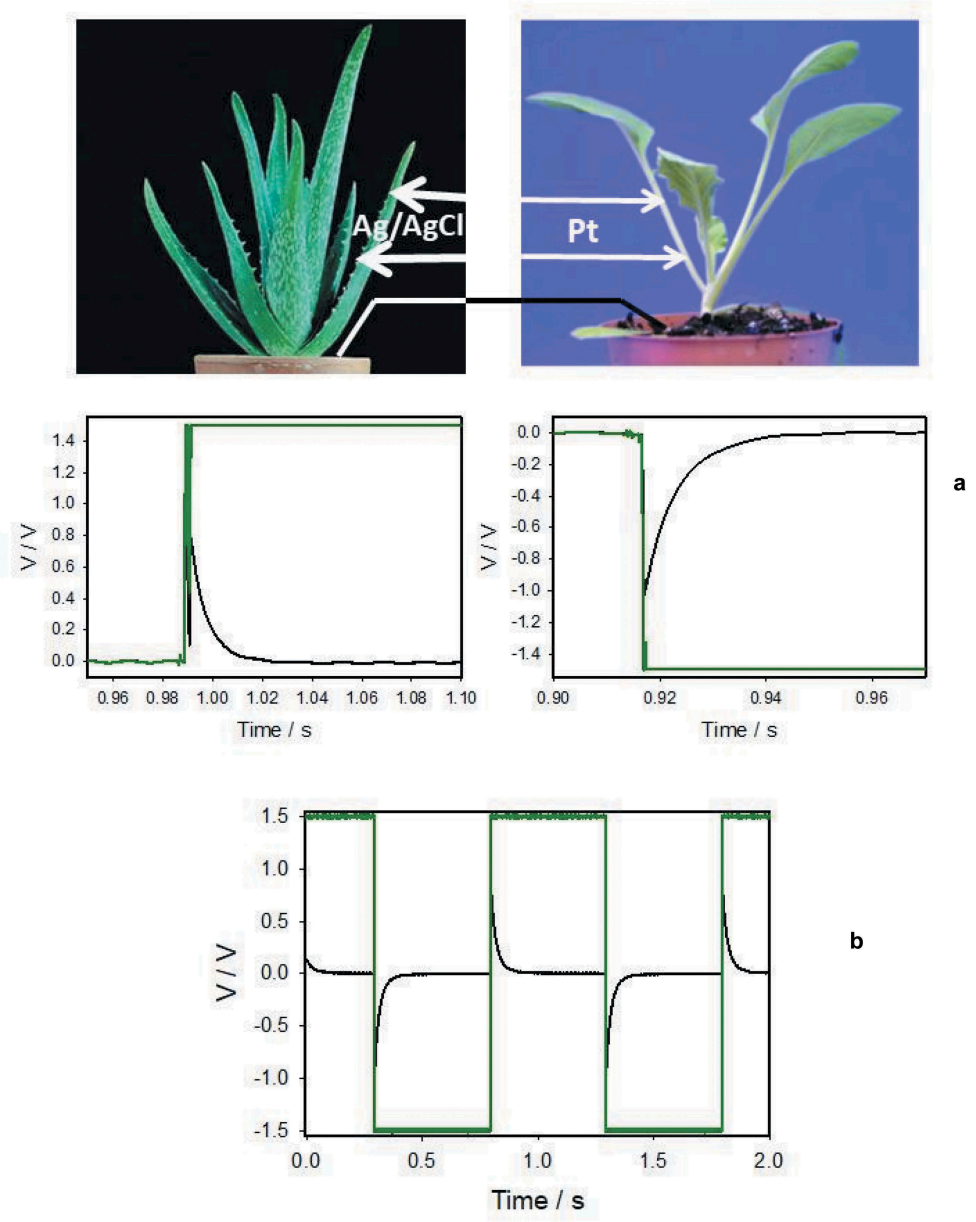
Electrical responses between Ag/AgCl electrodes in an *Aloe vera* leaf induced by 1.5 V electrical battery (green line) (a) or a function generator (b) connected to Pt-electrodes inserted to a neighboring cabbage plant. Distance between Pt-electrodes was 0.2 cm and distance between Ag/AgCl electrodes was 2 cm. Both pots with plants located at 10 cm distance were connected by a silver wire. Both of the ends of a silver wire were covered by electrodeposition of AgCl on 10 mm long wire tips without PFA coating.

Amplitude of electrotonic potential depends on frequency of sinusoidal wave applied for electrostimulation of plants. Amplitude of electronic potentials increases to a maximum amplitude with increasing of applied voltage frequency, and decreases at very high frequencies [2–4]. There is no electrotonic signal transmission between plants in the absence of electrical conductors between soils or plants in both pots. It proves that the fast electrotonic signal transmission between neighboring plants is not caused by volatile organic compounds’ emission, mycorrhizal networks in the soil, the plants’ rhizosphere, naturally grafting of roots, and acoustic communication. If one of plants is substituted by the *Aloe vera*, tomato or cabbage plants, results on electrostimulation and transmission of electrical signals look very similar.

There are different pathways for electrical communication within and between plants such as cell-to-cell, root-to-root, shoot-to-shoot, and between roots and shoots.

For electrostimulation, we used the pulse train, sinusoidal and triangular saw-shape voltage profiles. The amplitude and sign of passive electrotonic potentials depend on the amplitude, rise and fall of the applied voltage. Electrostimulation by a sinusoidal wave from a function generator induces electrical response between inserted Ag/AgCl electrodes with a phase shift of 90° at low frequencies of electrostimulation. The phase shift decreases at high frequencies of electrostimulation. This phenomenon shows that electrical networks in plants have electrical differentiators in cell-to-cell coupling. Electrical differentiators were found in *Arabidopsis thaliana*, cabbage, *Aloe vera, Mimosa pudica*, tomato plants, and in the Venus flytrap [2–4,16]. Cell-to-cell coupling in plants is well supported in the literature [12].

The sign of an electrotonic response depends on the polarity of electrostimulating electrodes and the amplitude of electrotonic potentials depend on the amplitude of applied voltage. The response does not obey the “all-or-none rule.” It is not an action potential but rather corresponds to the propagating electrotonic potential.

The equivalent electrical circuit of generation, underground transmission, and recording of electrotonic potentials between plants [2–4] can be extrapolated to electrical signal transmission in the plant-wide web.

Plants have developed complex systems of communication. Electrical, mechanical, and chemical signals induced by above-ground stresses in plants can affect below ground communication between roots of neighboring plants. There are different electrical, chemical and electrochemical pathways for underground signaling between plants. Electrical signal transmission is fast in comparison with chemical signaling which is controlled by a slow diffusion. Electrostimulation of plants induces electrotonic potentials transmission in the electro-stimulated plants as well as the neighboring plants located in different pots regardless if plants are the same or different types. Experimental results displayed cell-to-cell electrical coupling and the existence of electrical differentiators in plants. Electrostimulation serves as an important tool for the evaluation of mechanisms of communication in the plant-wide web. The work on physiological responses to electrical signals from the first to the second plant is in progress.

## Materials and methods

Seedlings of Bonnie Best Cabbage (*Brassica oleracea* L.) were purchased from Bonnie Plant Farm (Union Spring, Alabama). *Aloe vera* L. plants with 20–35 cm leaves were grown in clay pots with sterilized potting soil. Temperature of air was 21°C. All experiments were performed on healthy adult specimens. Plants were exposed to a 12:12 hr light/dark photoperiod at 21°C. The average air humidity was 40% and the irradiance was 550–800 μmol photons m^−2^ s^−1^ PAR at plant level.

Identical Ag/AgCl electrodes were used as working and reference electrodes for measurements of potential differences in the plants. Both of the ends of a silver wire were covered by electrodeposition of AgCl on 10 mm long wire tips without PFA coating. Platinum electrodes for plant electrostimulation were prepared from PFA-coated platinum wires (99.99% purity; *A-M Systems, Inc., Sequim, WA, USA*) with a diameter of 0.076 mm.

**Figure 2.**
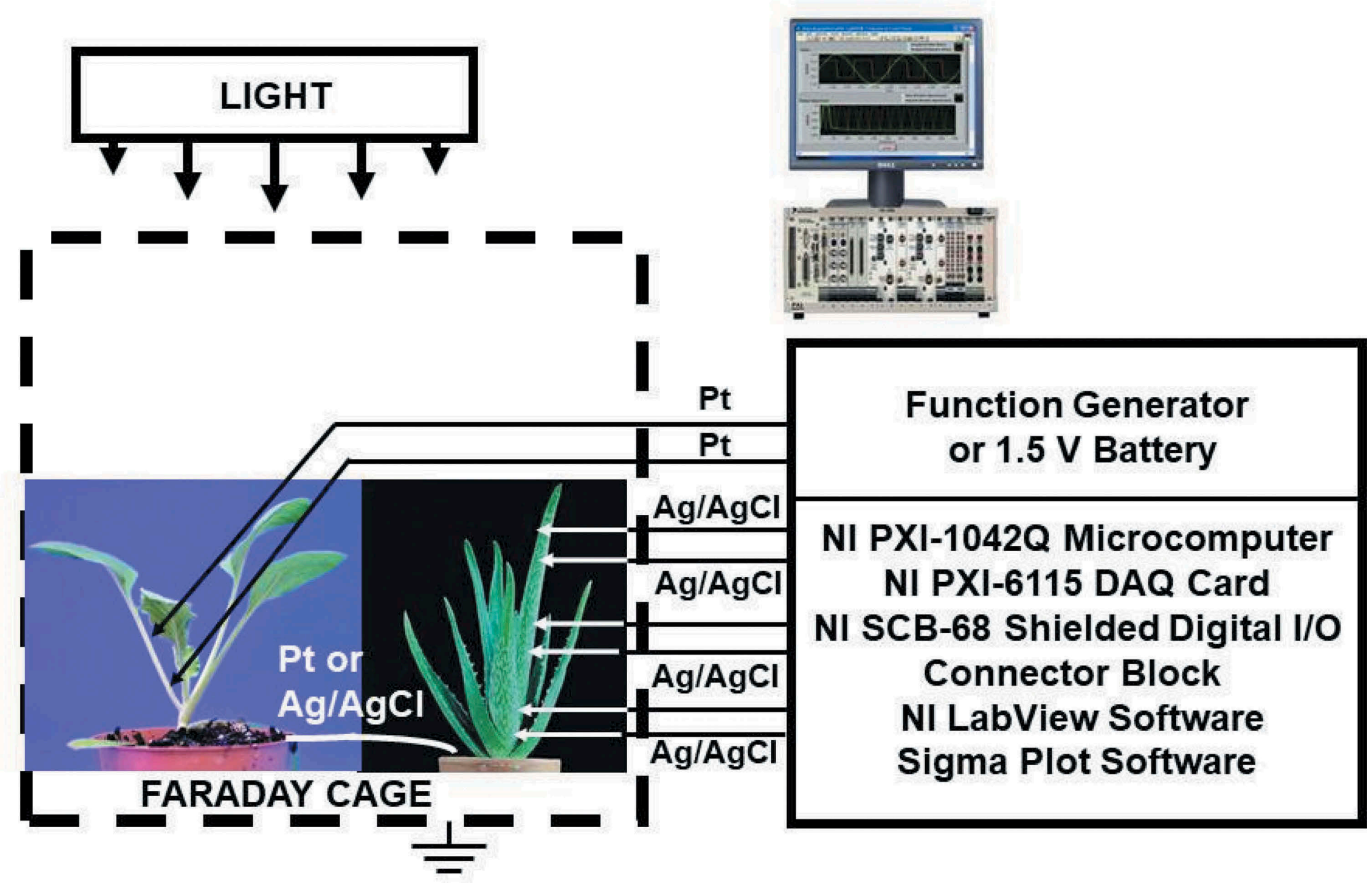
Experimental setup. Function generator or 1.5 V battery connected to platinum electrodes were used for electrostimulation of plants.

Two methods of plant electrostimulation were used: the function generator and the 1.5 V batteries. The function generator FG300 (*Yokagawa, Japan*) was interfaced to the NI-PXI-1042Q microcomputer and used for the electrostimulation of plants (Figure 2).

Note that the previously developed mathematical model [2–4] of electrotonic potentials transmission within and between tomato plants, which is supported by the experimental data, is generic enough to be used for simulation study and predicting the intercellular and intracellular communication in the form of electrical signals in the electrical networks within and between a variety of plants.
